# THE INFLUENCE OF NURSING HOME CHAMPIONS ON THE IMPLEMENTATION OF A PERSON-CENTERED QUALITY IMPROVEMENT PROJECT

**DOI:** 10.1093/geroni/igac059.1322

**Published:** 2022-12-20

**Authors:** Miranda Kunkel, Kamryn Kasler, Alexandra Heppner, Kimberly VanHaitsma, Katherine Abbott

**Affiliations:** Miami University, Oxford, Ohio, United States; Miami University, Oxford, Ohio, United States; Miami University, Oxford, Ohio, United States; The Pennsylvania State University, University Park, Pennsylvania, United States; Miami University, Oxford, Ohio, United States

## Abstract

Preferences for Activity and Leisure (PAL) Cards are a communication tool designed to alert nursing home staff to important resident preferences. This study explored the role of nursing home provider champions implementing (PAL) Cards in their community. Champions (n=35) created PAL Cards for 15-20 residents. A total of 88 monthly interviews were audio-recorded, transcribed, checked for accuracy, and coded using the domain “Characteristics of the Individual” from the Consolidated Framework for Implementation Research. Each construct was assigned a general valence rating based on consensus. Four constructs received the highest positive valence ratings and were identified as important facilitators to implementation success, and one construct received a neutral valence. Champions who anticipated the benefits of PAL Cards for their community and recruited a supportive team exhibited high implementation success. Additionally, the champion’s perception of help from staff seemed to increase their own self-efficacy to achieve their goals.

